# Sézary Syndrome and Atopic Dermatitis: Comparison of Immunological Aspects and Targets

**DOI:** 10.1155/2016/9717530

**Published:** 2016-05-17

**Authors:** Ieva Saulite, Wolfram Hoetzenecker, Stephan Weidinger, Antonio Cozzio, Emmanuella Guenova, Ulrike Wehkamp

**Affiliations:** ^1^Department of Dermatology, University Hospital of Zurich, University of Zurich, Gloriastrasse 31, 8091 Zurich, Switzerland; ^2^Department of Infectology and Dermatology, Riga Stradins University, Riga, Latvia; ^3^Department of Dermatology, Christian-Albrechts-University Kiel, Kiel, Germany

## Abstract

Sézary syndrome (SS), an aggressive form of erythrodermic pruritic cutaneous T cell lymphoma (CTCL), from an immunological perspective characterized by increased Th2 cytokine levels, elevated serum IgE and impaired cellular immunity. Not only the clinical appearance but also the hallmark immunological characteristics of SS often share striking similarities with acute flares of atopic dermatitis (AD), a common benign chronic inflammatory skin disease. Given the overlap of several immunological features, the application of similar or even identical therapeutic approaches in certain stages of both diseases may come into consideration. The aim of this review is to compare currently accepted immunological aspects and possible therapeutic targets in AD and SS.

## 1. Introduction

Sézary syndrome (SS) is a rare erythrodermic and leukemic variant of cutaneous T cell lymphomas (CTCL) that belongs to the heterogeneous group of extranodal non-Hodgkin's lymphomas (NHL) arising from the malignant proliferation of skin-homing T cells [1, 2]. SS together with mycosis fungoides (MF) are the most common forms of CTCL accounting for around 65% of cases whereas SS represent around 3% of all CTCL [3]. CTCL are assumed to have a male predominance and the median age at onset of the disease is between the fifth and sixth decade [4, 5].

The behaviour of the SS is aggressive with a median survival of 1–5 years [3, 6, 7]. SS and erythrodermic MF (E-MF), which is considered to be an advanced form of MF with absent or minimal blood involvement, may be referred to as erythrodermic CTCL (E-CTCL) [3, 8]. If blood involvement is present, the term leukemic CTCL (L-CTCL) is used and therefore it is applicable to every case of SS [1, 2]. Besides due to the lack of clear diagnostic markers the differential diagnosis of various erythrodermic skin diseases is still challenging [9]. Atopic dermatitis is a common chronic inflammatory skin disease with a lifetime prevalence of 15–20% in developed countries [10]. The majority of patients show an onset in early childhood and a remission until adolescence. However, recent prevalence estimates in adults of up to 10% indicate that the rate of persistent and/or adult-onset disease is higher than previously assumed [11, 12]. AD is an important differential diagnosis of SS in adults with erythrodermic dermatitis [10]. Although in majority of cases there are characteristics such as typical predilection sites for AD and palmoplantar hyperkeratosis for SS that allow clinically distinguishing between AD and E-CTCL, in some exceptional cases of erythroderma especially among the elderly population initially it might be a clinical challenge to define the diagnosis. The comparable clinical features are further reflected by some overlapping immunological peculiarities, in particular an epidermal barrier deficiency, and a cutaneous infiltration by CD4+ T helper cells expressing the skin-homing receptor cutaneous lymphocyte-associated antigen (CLA) and chemokine receptor 4 (CCR4). Interestingly, both AD and SS show increased production of Th2 cytokines such as interleukin 4 (IL-4), interleukin 5 (IL-5), and interleukin 13 (IL-13) as well as CCR4-binding chemokines that is characteristic also of the acute phase of AD [13–15]. As a consequence of the epidermal barrier deficiency and the diminished Th1 and Th17 cell immunity, the skin of AD patients shows a less diverse surface microbiome and an increased susceptibility towards cutaneous colonization and infection with* Staphylococcus aureus* (*S. aureus*) [10, 15]. The skin microbiome has not yet been systematically examined in CTCL, but there are preliminary data indicating increased* S. aureus* colonization rates in MF and SS [16]. Both AD and SS benefit from topical barrier restoring and rather unspecific topical or systemic immunosuppressive treatment, although SS often shows slower and/or weaker responses [10, 17]. As insights into the precise molecular mechanisms and key immunological networks driving inflammation grow, summarizing the knowledge about immune responses in these Th2 cell-dominated diseases may potentially allow drawing conclusions about different markers and therapeutic targets in both of the diseases. The aim of this review is to compare the immunological aspects and therapeutic targets in AD and CTCL.

## 2. Clinical Characteristics of E-CTCL

**Figure 1:**
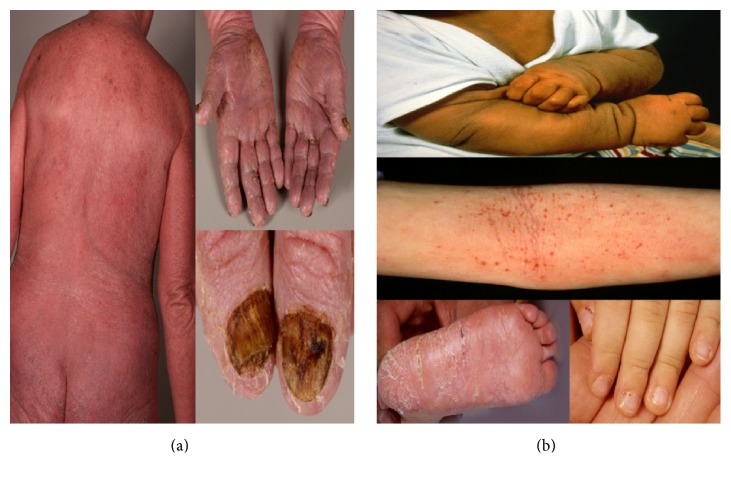
Clinical appearance of patient with Sézary syndrome (a) and atopic dermatitis (b).

SS is defined by a typical clinical triad consisting of erythroderma, peripheral lymphadenopathy, and peripheral blood involvement. Although in the majority of SS cases rapid onset of the clinical manifestations can be observed, in some patients a long medical history including disabling pruritus as well as nonspecific dermatitis is present. Cutaneous manifestations in E-CTCL comprise a broad clinical spectrum varying from mild erythema to generalized exfoliative erythroderma complicated by electrolyte dysregulation and high output cardiac failure due to the extensively dilated skin vessels [18, 19] (Figure 1(a)). Erythroderma is often accompanied by severe pruritus. Additionally, the patients may present with palmoplantar keratoderma and alopecia and nail changes varying from discoloration to subungual hyperkeratosis and ocular involvement, most frequently eyelid ectropion [20–22]. Elderly patients with erythrodermic eczematous pruritic skin may be a great clinical challenge for physicians with regard to differential diagnosis. Some case reports have described SS arising in patients with a long history of AD [23–26]. However, a study showed no significant difference in the prevalence of atopy in SS compared to MF and the general population [23].

To confirm the definite diagnosis in E-CTCL, clinicopathological correlation often including multiple skin biopsies with histopathological and immunohistochemical investigations and in most cases staging examinations (blood, lymph node, and other organs) are necessary [9, 27–29].

## 3. Clinical Characteristics of AD

The most characteristic features of AD are intense itch and recurrent eczematous skin lesions, which typically show an age-related morphology and distribution. Infants most often present with eczematous skin lesions on the cheeks and the scalp while in childhood predilection sites are the flexures, neck, and dorsal aspects of the limbs (Figure 1(b)). Starting with adolescence flexural areas, head and neck, shoulders, and hands are predominantly affected [30, 31]. The majority of patients display generalized skin dryness and a personal or familial history of atopy. Other associated features are a hyperlinearity of the palms and soles, Dennie-Morgan infraorbital folds, and Hertoghe's sign [10]. In general, AD shows a wide spectrum of clinical features and trajectories, in particular in adulthood, where the disease often presents as eczematous erythroderma and appears to show a male predominance [31, 32]. Adult-onset AD often shows an untypical distribution and morphology of lesions and is not well captured by classical diagnostic criteria and appears to be less closely associated with atopy as compared to early-onset forms, although reliable clinical and epidemiological data are lacking [10].

## 4. Immunological Aspects

**Figure 2:**
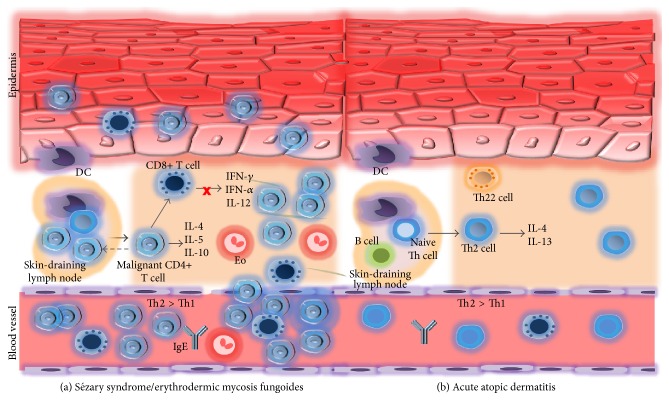
Th2 prevailing immune response in Sézary syndrome (SS)/erythrodermic MF (E-MF) and atopic dermatitis (AD). (a) SS/E-MF with malignant T cells circulating in the blood promote an immune response with production of Th2 phenotypic cytokines. Therefore suppressing the Th1 immune properties leads to impaired host immune response. (b) In the acute phase of atopic dermatitis naive T helper cells are primed in Th2 cells under the influence of activated skin resident DC which have the capacity of migrating to skin-draining lymph nodes.* DC: dendritic cell, Eo: eosinophilic granulocyte.*

In both L-CTCL and acute AD there is a predominance of Th2 immune response [33, 34]. An overview is shown in Figure 2. The complexity of the T cell compartment is not fully understood, and the role of T cells in the regulation of chronic inflammatory processes in skin diseases as well as in cancer is puzzling and yet to be defined.

The pathophysiological mechanisms underlying both entities, the one being a benign inflammatory skin disease, with the other representing a hematological malignancy of the skin, will be described with a focus to enlighten the similarities and differences for both conditions.

### 4.1. Distinct Features of Pathogenesis of SS (L-CTCL) and AD

#### 4.1.1. Malignant T Cells in CTCL

A cross talk between different cells of the innate immune system, benign bystander T cells, and malignant T cells is crucial for the immune responses in CTCL.

In initial MF skin lesions, an increased number of CD8 cytotoxic cells, in addition to FOXP3 regulatory T cells (Tregs), have been detected, which was attributed to an antitumor response promoted by dendritic cells [35–38]; on the contrary, the disease progression of MF was found to be associated with a remarkable decrease of normal Treg and cytotoxic T cells [39]. The exact role of Tregs in CTCL still remains controversial. Malignant T cells which, in some SS patients, exhibit phenotype characteristics for Tregs may contribute to the downregulation of the local antitumor response as well as systemic immunosuppression [37, 39, 40]. Furthermore, it has been suggested that SS might be a malignancy of FOXP3+ (forkhead box P3) regulatory T cells, Th2 cells, and Th17, whereas the presence of Th17 in another study could not be confirmed [33, 41–43].

Several studies of peripheral blood mononuclear cells have demonstrated increased production of Th2 cytokines and reduced production of Th1 cytokines in L-CTCL [44, 45]. Gene expression studies confirmed elevation of Th2 associated genes in the PBMC and skin of patients with L-CTCL [46, 47]. The association of increased IL-4 production with advanced stage L-CTCL suggests that the source of excess Th2 cytokines may be the malignant clone itself.

This hypothesis has recently been corroborated by studying the functional bias of malignant T cells in L-CTCL patients in whom the malignant T cell clone could be conclusively identified by staining with antibodies against TCR V *β*. Flow cytometrical assessment of the intracellular cytokine production demonstrated that both the malignant clone and surprisingly the remaining benign T cells expressed high levels of the Th2 cytokines IL-4 and IL-13 and negligible levels of IFN-*γ* upon activation [33].

Several recent studies were dedicated to the origin of the malignant T cells. Interestingly, malignant T cells, isolated from the skin of SS patients, showed not only CCR7 and CCR4 but also high expression of L-selectin and CD27, a phenotype characteristic of central memory T cells [1]. On the other hand absence of CCR7/L-selectin and CD27 expression has been shown in T cells from MF lesions, whereas CCR4 and CLA are highly expressed suggesting a phenotype of skin resident effector memory T cells [1]. These findings led to the hypothesis that SS is a malignancy arising from central memory T cells, while MF is a malignancy of skin resident effector memory T cells, supporting evidence that SS and MF should be considered as separate lymphomas originating from distinct functional T cell subsets [1].

In 2004 Rübben et al. proposed the intriguing hypothesis that CTCL arises from genetically unstable subclones that undergo a multilineage progress into a stable clone leading to subsequent proliferation of neoplastic T cell population that originates MF [48].

In general, progression of the disease correlates with a decrease of the T cell receptor (TCR) repertoire, paralleled by a predominance of clonal, malignant CD4+ T cell population, expressing a single TCR clone [49].

#### 4.1.2. T Cells in AD

Regarding immune dysregulation in AD, it is known that in acute phase of AD a dense infiltrate of CD4+ cells as well as allergen specific CD4+ and CD8+ T cells can be found in the affected skin lesions, thus promoting cutaneous inflammation [50].

During an acute onset of the disease Th2 bias is characteristic whereas along with the chronification an increasing proportion of the skin is being infiltrated by Th1 cells accompanied by Th2, Th0, and Th22 cells [13, 51–54].

In AD naive T helper cells are being polarized into Th2 phenotype by activated skin resident dendritic cells that have migrated to local lymph nodes [52, 55].

The hallmark Th2 cytokines IL-4 and IL-13 play important role in the pathogenesis of AD. Following IL-4 dependent induction of IgG class switch in B-cells, a subsequent elevation of IgE levels can be frequently observed in AD patients [56]. Furthermore, IL-4 is important for the functional phenotype of immigrating DC precursors and thereby heavily influences the phenotype of an ongoing immune response [34].

Thymus and activation regulated chemokine (TARC/CCL17), a member of the Th2 chemokine family, serum level has been suggested to be a useful clinical biomarker for AD treatment and disease severity [57, 58], whereas in CTCL at disease progression chemokine receptors, expressed by skin-infiltrating T cells and surface molecules, have shown the tendency to decrease due to loss of these markers subsequently followed by diminished epidermotropism [59, 60].

However in contrast for chronic AD lesions there is an increase of Th1 cytokines: interferon-*γ*, IL-12, IL-5, and GM-CSF [61, 62]. Recently, it has been suggested that in Th2 cell mediated dermatitis persistence of chronic inflammation might be induced by TLR2 ligands through IL-4-mediated suppression of IL-10 [63].

#### 4.1.3. Factors Contributing to Impaired Cellular Immunity in CTCL

In addition, many other mechanisms might be responsible for the inhibition of cellular immunity by CTCL such as dysregulated expression of the immunoregulatory proteins (e.g., CTLA-4, PD-L1 (programmed-death-ligand 1), and Fas ligand (Fas L)) and constitutive activation of Jak/Stat pathway, which promotes transforming growth factor *β* (TGF-*β*) and IL-10 secretion [43, 64–66]. Programmed-death-receptor-1 (*PD-1*), a membrane molecule of CD28/CTLA-1 receptor family, plays an important role in cellular immunity. By interaction with its ligands it has been shown to inhibit T cell activation and proliferation [67]. It has been demonstrated to be highly expressed by neoplastic T cells in SS [68]. Although its role in pathogenesis of CTCL is not clear, it might contribute to the immunosuppression in SS [68, 69].

*Defective apoptosis* has been shown to be characteristic of SS. It has been demonstrated to correlate with decreased or impaired death receptor mainly Fas expression by neoplastic T cells, resulting in perpetual neoplastic T cell proliferation [70–75].

Further it has been shown that* adhesion molecules and chemokines* have a significant contribution to skin-homing of malignant T cells in CTCL. The mechanisms are not yet fully elucidated, but some factors that promote T cells prone to skin-homing, for example, adhesion molecules and chemokines, have been identified. Among them are cutaneous lymphocyte-associated antigen (CLA), chemokine receptor 4 (CCR4), CCR10, and CCR7 whose expression has been demonstrated by malignant T cells in patients with MF and SS [76–82]. CXCR4, a receptor for CXCL12, which can also be observed in SS, may contribute to cell skin recruitment and accumulation through the regulatory activity of CD26 in CTCL [83, 84]. The mechanisms are not yet fully elucidated, but some factors that promote T cells prone to skin-homing, for example, adhesion molecules and chemokines, have been identified [77, 82, 83, 85].

#### 4.1.4. Aspects Leading to Impaired Skin Barrier in AD

The major hallmarks in the pathogenesis of AD are an impaired epidermal skin barrier function and an immune dysregulation. Genetic background with filaggrin protein gene mutations along with other factors such as skin cytokine imbalance leads to decreased filaggrin expression, which belongs to one of the most crucial factors underlying the epidermal barrier dysfunction in AD [10, 31]. Moreover, filaggrin deficiency has been associated with subclinical inflammation, reduced resistance to irritants and haptens, and an enhanced percutaneous allergen sensitizing [10]. Another peculiarity in AD is decrease and alterations in lipids such as ceramides of the stratum corneum. Besides keratinocytes not only maintain the first line of physical barrier but also express pattern recognition receptors for several agents including proteolytic allergens that are also capable of inducing Th2 cell mediated immune responses [10].

Several host and environmental aspects contribute to the epidermal barrier dysfunction in AD that increase skin penetration for allergens, microorganisms, and irritants.

### 4.2. Joint Aspects of Pathogenesis of SS (L-CTCL) and AD

#### 4.2.1. Th2 Weighted Immune Response

Although in early stages of MF a dominance of Th1 immune answer is prevailing, a later progression of the disease reveals an immune response that is dominated by Th2 malignant T cells. Th2 weighed immune response is characteristic of an acute phase of atopic dermatitis as well [18, 30, 33, 44].

In SS, a Th2 weighed immune response is present with an overproduction of the typical cytokine profile, IL-4, IL-5, IL-10, and IL-13, respectively, and additionally elevated levels of serum immunoglobulin E (IgE) and immunoglobulin A (IgA) and peripheral eosinophilia [33, 42, 44, 86]. Along with the disease progression, a decline in the number and activity of benign immune cells results in an impaired cell mediated cytotoxicity and decreased antigen-specific T cell responses and consecutively in a severe immunodeficiency [17, 33]. Interestingly, in SS patients a global Th2 bias with enhanced production of Th2 cytokines has been shown to be characteristic of both benign and malignant T cells. Th2 bias was demonstrated to be intrinsic in malignant T cells but extrinsic in benign T cells. It has been demonstrated that the Th2 cytokines from malignant cells are capable of inhibiting the Th1 responses [33].

Atopic Dermatitis Is Also Th2 Prototypic Disease in the Acute Phase. Consequently, in the acute phase a predominance of Th2 cytokines, IL-4, IL-5, and IL-13, is observed; therefore with regard to cytokine profile some parallels between AD and L-CTCL can be drawn.

This Th2 phenotype shift may be an important factor prompting an infectious susceptibility observed in patients with SS as well as AD [14, 75, 76]. A better understanding of these interactions in SS and AD would promote further targets for treatment.

#### 4.2.2. Overlapping Factors That May Promote Th2 Weighed Immune Response in Both AD and SS

Eotaxins, chemokine ligands (CCL) CCL11 and CCL26 that are expressed by epidermal keratinocytes and dermal fibroblasts support the chemotaxis of Th2 cells positive for chemokine receptor 3 (CCR3) and eosinophils, thereby also promoting the Th2 bias [39, 87–90]. There is an increased expression of CCL11 and CCL26 both in serum and in lesional skin in AD patients therefore suggesting that it might also play an important role in the pathogenesis of the disease [91, 92]. Also in patients with advanced MF/SS elevated CCL11 and CCL26 levels in comparison with healthy individuals have been demonstrated [90].

In addition, elevated serum levels of TSLP that activates immature myeloid dendritic cells (DC) to produce CCL17 in CTCL patients were detected. Also in AD high expression in keratinocytes in affected skin lesions was seen and TSLP might promote leading towards a Th2 phenotypic immune response [55, 93–95].

### 4.3. Joint Aspects of Pruritus in AD and SS (L-CTCL)

Several receptors, secreted molecules (histamine, nerve growth factor (NGF), and substance P (SP)), proteases, and cytokines/chemokines (thymic stromal lymphopoietin (TSLP), IL-2, IL-4, IL-13, and IL-31) along with many others are described as contributing to chronic pruritus [96]. Although the role of distinct players in AD is ambiguous, IL-31 strongly contributes to pruritus in AD also correlating with the severity of the disease [97, 98]. Interestingly, some studies have suggested that there is no general relationship between IL-31 protein expression and pruritus in Th2 weighted diseases. Therefore, IL-31 might play a distinct role in the pathogenesis of AD [17].

Although tumor cells in MF/SS exhibit similar cytokine profile with AD and both are characterized with pruritus, the analysis of the IL-31 pathway in MF/SS patients regarding serum levels as well as receptor expression does not suggest a central role of IL-31 in MF/SS pathogenesis. Nevertheless increased levels of IL-31 were observed in patients with severe pruritus; therefore it might be a rationale for therapeutic approach for some patients [99].

### 4.4. Joint Factors of Immune Dysregulation Leading to Increased Infectious Susceptibility in SS and AD

Regarding atopic dermatitis it is still a matter of debate whether the primary factor is the disturbed immune response that results with a defective epidermal skin barrier function or* vice versa*. Nevertheless, there is no doubt that both components have a strong contribution and there is a close interaction among them; moreover once the inflammatory cascade has been activated they both belong to the* Circulus vitiosus*.

Keeping in mind that acute AD and L-CTCL are both characterized by Th2 phenotypic immune response one would expect some overlap in clinical consequences as well.

In both AD and L-CTCL Th2 cytokines modulate the immune response and lead to defective cutaneous barrier function by impairing keratinocyte protein differentiation and downregulating the antimicrobial peptides in the skin and therefore the innate immunity of the skin. Both the immune dysregulation and the decreased skin barrier predispose to an extensive bacterial colonization and increased risk of skin infections, which can be observed in patients with AD and SS as well [100–102].

Whereas for both AD and L-CTCL the immune dysregulation promotes the increased susceptibility to infections, in case of L-CTCL this aspect appears to play the major role.

As already mentioned above, there is reduced skin microbiome diversity among AD patients with a prevailing colonization of* Staphylococcus aureus* (*S. aureus*). In patients with MF and SS, there likewise seems to be an overabundance of staphylococcal carriage to a similar extent as in AD [16, 103–105].

Besides IgE antibodies against* S. aureus* toxins have been shown to exhibit superantigen properties, which appear to correlate with the severity of the disease [106]. In addition,* S. aureus* toxins themselves exhibit superantigen properties [107]. Moreover, it has been shown that staphylococcal enterotoxin B (SEB) upregulates IL-31 in peripheral blood mononuclear cells [108, 109]. It is assumed that signal transducer and activator of transcription 3 (Stat3) and the immunoregulatory cytokine interleukin 10 (IL-10) may play an important role in immune dysregulation in CTCL. During disease progression malignant activation of Stat3 and expression of IL-10 increase in parallel with the evolving immune impairment [42, 65, 110, 111].

Furthermore, it has been shown recently that microbial toxins, namely, staphylococcal enterotoxins, might be part of vicious circle not only as an epiphenomenon but also by stimulating benign T cells to induce activation of the immunoregulatory Stat3/IL-10 axis in malignant T cells. Accordingly in CTCL colonization with S. aureus that produces enterotoxin may promote the skin immune dysregulation [112].

Eradication of pathogen skin microflora has been associated with clinical improvement by several authors [16, 104, 112, 113].

Furthermore, therapies that inhibit Th2 cytokine activity, hence keeping a balance of Th1 responses, may have the potential to enhance both antipathogen and antitumor responses [16, 33, 114, 115].

## 5. Therapy

In both SS and AD there is a repertoire of standard therapies that is used according to current guidelines in respect of the extent of skin and systemic involvement [116–119]. These standards will not be discussed in this review. In the past years newly developed therapeutic agents have led to a dramatically improved therapeutic outcome and change of treatment rationale for many diseases. This is particularly true for cancer therapy and in dermatology revolutionizing the treatment for melanoma implementing targeted therapy. The aim of targeted therapy is the destruction of tumor cells and induction of as few side effects as possible. Due to the complexity of cancer and having no exclusive target markers on cancer cells this concept comes not always true but nonetheless has led to an immense advancement and better understanding of the pathophysiology in many diseases. Targeted therapies have been developed not only for cancer but also for inflammatory diseases. Despite the efforts in developing effective treatments in CTCL until today, apart from stem cell transplantation cure is not achievable [120]. However, induction of partial or complete remission is possible [121].

In the following, we focus on immunologic checkpoints that are of interest in SS and are currently implemented in clinical studies. We compare the checkpoints to data drawn from studies in AD and/or evaluate potential benefits from transferring therapeutic approaches from one entity to the other. The list of targets mentioned here is not exhaustive.

All information about ongoing studies without published results was obtained at the official homepage of the registry and results database for clinical studies in human participants: https://clinicaltrials.gov/. An overview about the targets discussed in this review is given in Table 1. Detailed information and references are to be found in the text.

### 5.1. Targeting Structures of the Immune System

SS arises from the lymphocytic system, which is per se part of the immune system. The cell of origin is a central memory T cell with the ability of circulation in skin, blood, and lymph nodes [1]. Therefore, targeted therapy for SS always means interacting with immune mechanisms.

In AD multiple factors seem to contribute to this condition such as underlying genetic predisposition and environmental factors. Excessive T cell activation is characteristic of AD with still unclear exact pathophysiologic mechanisms [122]. Targeting the T cells and/or the environment of neoplasm/inflammation is a reasonable approach in both diseases. The aim-result of targeted therapy is either induction of immunotoxic effects on cancer cells or modification of immunological mechanisms. The latter is the more common in the currently developed agents. In malignant T cell proliferation blocking immunosuppression or enhancing the local immune response is supposed to be beneficial. Many targets to act on these differing approaches are under exploration.

### 5.2. Targets on T Cells

*Alemtuzumab* is a humanized monoclonal anti-CD52 directed antibody. CD52 is described as a pan-T- and B-cell-marker, expressed also on dendritic cells and macrophages. Alemtuzumab has proven to be especially useful in SS and MF with leukemic involvement, which seems to be related to the cell of origin of the clonal malignancy and its surface marker profile [1, 123]. It leads to a depletion of circulating lymphocytes and thereby suppression of immune responses [124]. Application may result in severe adverse events, mainly infections [125]. Its use in AD has to be discussed carefully. There is no data published on this topic.

*Ipilimumab (Yervoy®)* is a monoclonal antibody targeted against CTLA-4. CTLA-4 obviously is overexpressed in CTCL and SS and coincides in the latter with abnormal findings for IL-10 and Foxp3 [126]. This phenotype would suggest a differentiation towards Tregs [41]. Blocking CTLA-4 might therefore be an interesting therapeutic approach. However, there is no data yet on CTLA-4 inhibition in CTCL and the exact mechanism of action has not yet been completely elucidated. Overexpression of CTLA-4 in CTCL could be interpreted as feature either of an immune escape mechanism or potentially of immunosuppression [127]. In Hodgkin lymphoma a study for exploration of the combinational therapy of ipilimumab with brentuximab vedotin and nivolumab has been initiated. The three agents are used in three different arms comparing nivolumab + brentuximab vedotin versus ipilimumab + brentuximab vedotin versus the combination of all the three drugs. Transferring the results of this study will be highly interesting for the treatment of CTCL, especially CTCL with CD30 expression.

*PD-1 and programmed-death-ligand 1 (PD-L1)* are immunologic checkpoints on T cells and cancer cells, respectively, and their therapeutic blockage has led to convincing results for overall survival improvement in non-small-cell lung cancer, melanoma, and other solid tumors [128]. Approved in melanoma and non-small-cell lung cancer in Europe are two PD-1-inhibitors* pembrolizumab (MK-3475/Keytruda®)* and* nivolumab (Opdivo®)*. A phase 2 study with pembrolizumab for the treatment of relapsed/refractory MF/SS is active. Estimated primary completion date is January 2018. PD-L1-inhibitors are not yet approved but being tested in solid tumors and systemic lymphoma. Though not yet studied in CTCL, they probably will be in near future.

CCR4 is the receptor for CCL17 and CCL22 and is expressed mainly on CD4+ T cells with Th2 polarization. It can also be detected on other cells of the immune system like macrophages and dendritic and NK cells [129]. In SS CCR4 expression could be observed in the peripheral blood and in the skin [77].

*Mogamulizumab (Poteligeo®) (Anti-CC chemokine receptor 4 (CCR4))* is a new humanized monoclonal antibody obtainable in clinical studies for use in CTCL and SS and is currently in a phase 3 study in comparison with the HDAC inhibitor vorinostat (Zolinza®). In a phase 1/2 study in patients with CTCL, the subgroup of 19 SS patients showed the highest overall response rates with 47.1% [130]. Mogamulizumab leads to a depletion of CCR4+ malignant T cells and CCR4+Tregs. This mechanism is of great potential in T cell lymphoma therapy [131]. As mentioned above T cells are CCR4+ in both AD and SS [132]. Whether the depletion of Tregs via the CCR4 receptor might be beneficial in atopic patients has yet to be shown. A phase 1 trial has been conducted in patients with asthma. The results are still being evaluated.

### 5.3. Immunologic Targets in the Environment

*Interleukin 12 (IL-12)* is a promising target in CTCL and inflammatory skin diseases. In pediatric AD patients IL-12 levels in serum have been reported to be high [133]. Interestingly, the IFN serum levels, which would be expected to increase by stimulation through IL-12, are comparably low, leading to the assumption that the normal IL-12/IFN pathway is not intact [134]. Reduced IL-12R beta (2) mRNA expression may be the cause of low IL-12 receptor expression. High IL-12 without a binding receptor would have no cellular effects and would explain the low IFN serum levels [135]. IL-12 substitution would in this case not be beneficial for AD patients. In CTCL induction of lesion regression and cytotoxic T cell responses have been described under IL-12-therapy [136]. A phase 2 study with NM-IL-12 (recombinant human IL-12) should start in November 2015 as a single arm, open-label, nonrandomized study with NM-IL-12 (150 ng/kg) dosed in combination with low dose total skin electron beam (TSEB) in CTCL patients including patients with SS. IL-12 in this setting acts as immunotherapy to increase antitumor efficacy against CTCL, supposedly reducing skin-related toxicity.

*IL-13* has just recently been described as a contributor to growth of tumor cells in CTCL. IL-4 and IL-13 seem to act synergistically in this setting [137, 138]. Based on this detection, blockage of IL-13 receptor might be beneficial. An IL-13-R-inhibitor is available in experimental studies under the name* lebrikizumab (TNX-650)*. The drug has been used in a phase I study in Hodgkin's lymphoma and is currently under investigation in a phase 2 study in AD. Results for these two studies are not yet published. For asthmatic patients the application of lebrikizumab showed improvement and was considered as safe and of good tolerability [139]. Another IL-13-R-inhibitor that is in use in clinical studies is* tralokinumab*.

As mentioned above the synergism between IL-4 and IL-13 seems to contribute to tumor cell growth. With regard to this, targeting IL-4 or IL-4-receptor, respectively, would be another therapeutic option. A human monoclonal antibody against the IL-4-receptor (IL4R),* dupilumab*, revolutionizes the therapy in AD [140–142]. It is directed against the shared alpha subunit of the IL4R and, by IL4R blockage, it modulates signaling of both the IL-4 and IL-13 pathway. Exploration of IL-4 in SS showed a significant elevation of IL-4 positive cells compared to inflammatory dermatoses [143]. This finding would support the rationale for treating CTCL patients, especially SS patients, with IL-13/IL-4 inhibitors.

*Resimmune® (*or* A-dmDT390-bisFv(UCHT1) Immunotoxin) (Angimmune LLC)* is a recombinant immunotoxin selectively targeting the CD3 receptor and temporarily depleting all T cells. It has been shown that sensitivity of malignant T cells to this drug is 30 times higher compared to normal resting T cells. The drug may have an immunomodulatory effect by activating novel naive T cells contributing to further deletion of residual tumor cells [144]. Resimmune has been investigated in a clinical phase 2 study in patients with CTCL, including patients with SS. The results are not yet published.

Ten different types of* toll-like-receptors (TLR)* have been described in human until today. Their localization on antigen-presenting plasmacytoid and myeloid dendritic cells and their role in the immune response in cancer render them attractive targets for treatment. Apoptosis of tumor cells (e.g., under radiotherapy) and treatment with TLR may lead to synergistic effects [145, 146].* TLR7 agonist (imiquimod/Aldara®)* is available for topical application and is approved in Europe for treatment of superficial basal cell carcinoma and actinic keratoses. Its effects have also been shown for CTCL patients with response rates up to 50% [147–149].* TLR9 agonist (CPG 7909)* has been applied subcutaneously to SS and MF in a phase I study enrolling 28 patients. Clinical response rate was 32% [150]. In another study, TLR9 agonist was injected intralesionally and combined with radiation in 14 MF patients leading to the immunological effect of an in situ vaccination. The overall response rate was 35.7% [151]. In mouse the TLR9 agonist was combined with ibrutinib, which is a Bruton-tyrosine kinase-inhibitor. This approach led to an enhancement of the antitumor response [152]. This effect of the combination will have to be evaluated in humans and maybe become a therapy option in the future.

*Anti-CXCR4* is a potential treatment option for patients with SS. Because of CXCR4 overexpression in SS and MF, clinical studies for this target would be interesting [84]. For systemic lymphoproliferative diseases like acute myeloid leukemia the antibody* plerixafor (Mozobil®)* is in use. Though there is not yet a study running in CTCL, exploration of this therapy in near future is very likely.

*IL-22* is produced by T cells. Primarily its role in inflammation in human skin has been in focus, for example, in AD, but obviously it is also involved in the pathogenesis of malignant skin proliferation [153]. In CTCL elevated levels of mRNA and protein levels were detected for IL-22 [154].* Fezakinumab (ILV-094)*, a monoclonal antibody against IL-22, is being investigated in a phase 2 study in AD. Experimental use in CTCL could give interesting results.

Another interleukin that could be important in signaling in CTCL is* IL-18*. IL-18 has been interrelated to linking inflammatory immune responses and tumor progression [155]. In skin lesions increased IL-18 expression has been detected and potentially contributing to the elevation to serum levels in patients with CTCL and cutaneous NK cell lymphoma [156, 157]. High levels of IL-18 have been described in other skin malignancies as well. This might therefore be either a shared mechanism in skin tumorigenesis or part of an immune response mechanism [158]. Assuming that high levels of IL-18 are beneficial in cancer or lymphoma, respectively, human recombinant IL-18 (SB-485232) was administered in combination with rituximab in a phase 1 study to patients with non-Hodgkin's lymphoma (NHL) revealing a response rate of 26% [159]. Phase 2 studies in NHL are ongoing. Further workup is needed in patients with SS to clarify the role of IL-18 and to be able to evaluate if patients would profit from this treatment. In AD serum levels for IL-18 are found to be elevated likewise [160, 161]. Whether IL-18 application would be valuable for the patient is discussed controversially. Some authors propose that IL-18 might trigger the development of skin lesions in AD [162].

### 5.4. Others

In addition to all of the above-mentioned treatments in CTCL, there are many more that may have, not directly but indirectly, effects on the immune response. Epigenetic modifiers like histone deacetylase (HDAC) inhibitors or substances that interact with JAK/Stat or NF*κ*B may have influence on disease evolution. Furthermore, there are proteasome inhibitors like* bortezomib (VELCADE®)* that have been used in SS and targeted therapy directed against molecules expressed on cancer cells and linked to cytotoxic agents like the already well-established anti-CD30 antibody-drug conjugate* brentuximab vedotin (Adcetris®)* [163]. Expression of CD30 is not exclusively restricted to cancer cells, though there are only few cell types in a healthy individual with CD30 surface markers [164]. Tyrosine kinase inhibitors like* dasatinib* have been developed. B-lymphoid tyrosine kinase is a designated oncogene that could be targeted by dasatinib in CTCL [165]. A study has been conducted in metastatic and/or not surgically removable lesions in various types of lymphoma, including CTCL and SS. Results have not yet been published.

## 6. Conclusion

Overlapping features in immune responses in both diseases might be used for transferring knowledge for target molecules from one entity to the other.

A better understanding of the underlying mechanisms and new markers will hopefully yield further improvement of targeted treatment options in both AD and SS. Several promising diagnostic and clinical markers and new treatment options targeting checkpoints in the immune system have already been discovered and are implemented in clinical studies both for SS and for AD. In SS, there is still a need for efficacious therapy. Blocking the immunosuppressive mechanisms orchestrated by malignant T cells as well as enhancing the local immune response could be beneficial. Further exploration is necessary to prolong the life of patients and improve their quality of life.

## Figures and Tables

**Table 1:** Current and potential immunologic therapeutic targets in Sézary syndrome/cutaneous T cell lymphoma and atopic dermatitis in alphabetical order.

Target^*∗*^	Substance	Phase	NCT number (https://clinicaltrials.gov/)	Indication SS/CTCL	Indication AD	Approved for (in Europe)	Comments (more information & references in text)
CCR4	Mogamulizumab (KW-0761)	3	NCT01728805	X			Current study in comparison to HDAC inhibitor
CD3	A-dmDT390-bisFv(UCHT1) Immunotoxin	2	NCT00611208	X			Not likely to be used in AD
CD25	Denileukin diftitox	na		X		CTCL	Not likely to be used in AD
CD52	Alemtuzumab	na		X		Multiple sclerosis	Had been withdrawn from the market for CTCL and newly approved for multiple sclerosis (no medical reasons), not likely to be used in AD
CTLA4	Ipilimumab	na				Malignant melanoma	Interesting in CTCL, less in AD, combination study with nivolumab, ipilimumab, and brentuximab in HL ongoing
CXCR4	Plerixafor	na					Used in AML, interesting for SS/CTCL
IL-4R	Dupilumab (REGN668)	3	NCT02277769		X		Interesting as well in CTCL
	Pitrakinra	2	NCT00676884		X		
IL-12	NM-IL-12	2	NCT02542124	X			In combination with TSEB
IL-13	Lebrikizumab (TNX-650)	2	NCT02340234		X		Has been studied in NHL in phase 1, data not yet available
	Tralokinumab	2	NCT02347176		X		
IL-18	SB-485232	na					In phase 2 in NHL, interesting in CTCL, controversial opinions for use in AD
IL-22	Fezakinumab (ILV-094)	2	NCT01941537				Potentially interesting in SS/CTCL
PD-1	Pembrolizumab (MK-3475)	2	NCT02243579	X		Both in malignant melanoma	Not likely to be used in AD, combination study with nivolumab, ipilimumab & brentuximab in HL ongoing
	Nivolumab	na					
PD-L1	Avelumab	na					In phase 1 in HL, interesting in SS/CTCL
TLR7	Imiquimod	na		X		Basal cell carcinoma, actinic keratoses, and genital warts	Topical application, leading to inflammatory reactions, not promising for AD
TLR9	CPG 7909	1	NCT00043420	X			Interesting because of subcutaneous application (no restriction to skin surface)

SS: Sézary syndrome, CTCL: cutaneous T cell lymphoma, AD: atopic dermatitis, HDAC: histone deacetylase, na: not active in SS/CTCL or AD, NHL: non-Hodgkin's lymphoma, AML: acute myeloblastic leukemia, TSEB: total skin electron beam, and HL: Hodgkin's lymphoma. ^*∗*^Full names of targets in the text.

## References

[1] Campbell J. J., Clark R. A., Watanabe R., Kupper T. S. (2010). Sézary syndrome and mycosis fungoides arise from distinct T-cell subsets: a biologic rationale for their distinct clinical behaviors. *Blood*.

[2] Clark R. A., Shackelton J. B., Watanabe R. (2011). High-scatter T cells: a reliable biomarker for malignant T cells in cutaneous T-cell lymphoma. *Blood*.

[3] Willemze R., Jaffe E. S., Burg G. (2005). WHO-EORTC classification for cutaneous lymphomas. *Blood*.

[4] Imam M. H., Shenoy P. J., Flowers C. R., Phillips A., Lechowicz M. J. (2013). Incidence and survival patterns of cutaneous T-cell lymphomas in the United States. *Leukemia and Lymphoma*.

[5] Bradford P. T., Devesa S. S., Anderson W. F., Toro J. R. (2009). Cutaneous lymphoma incidence patterns in the United States: a population-based study of 3884 cases. *Blood*.

[6] Scarisbrick J. J., Prince H. M., Vermeer M. H. (2015). Cutaneous Lymphoma International Consortium study of outcome in advanced stages of mycosis fungoides and Sézary syndrome: effect of specific prognostic markers on survival and development of a prognostic model. *Journal of Clinical Oncology*.

[7] Olsen E., Vonderheid E., Pimpinelli N. (2007). Revisions to the staging and classification of mycosis fungoides and Sezary syndrome: a proposal of the International Society for Cutaneous Lymphomas (ISCL) and the cutaneous lymphoma task force of the European Organization of Research and Treatment of Cancer (EORTC). *Blood*.

[8] Vonderheid E. C., Bernengo M. G., Burg G. (2002). Update on erythrodermic cutaneous T-cell lymphoma: report of the international society for cutaneous lymphomas. *Journal of the American Academy of Dermatology*.

[9] Klemke C. D., Booken N., Weiss C. (2015). Histopathological and immunophenotypical criteria for the diagnosis of Sézary syndrome in differentiation from other erythrodermic skin diseases: a European Organisation for Research and Treatment of Cancer (EORTC) Cutaneous Lymphoma Task Force Study of 97 cases. *British Journal of Dermatology*.

[10] Weidinger S., Novak N. (2016). Atopic dermatitis. *The Lancet*.

[11] Margolis J. S., Abuabara K., Bilker W., Hoffstad O., Margolis D. J. (2014). Persistence of mild to moderate atopic dermatitis. *JAMA Dermatology*.

[12] Silverberg J. I., Hanifin J. M. (2013). Adult eczema prevalence and associations with asthma and other health and demographic factors: a US population-based study. *Journal of Allergy and Clinical Immunology*.

[13] Biedermann T., Skabytska Y., Kaesler S., Volz T. (2015). Regulation of T cell immunity in atopic dermatitis by microbes: the Yin and Yang of cutaneous inflammation. *Frontiers in Immunology*.

[14] Suga H., Sugaya M., Miyagaki T. (2014). Skin barrier dysfunction and low antimicrobial peptide expression in cutaneous t-cell lymphoma. *Clinical Cancer Research*.

[15] Kezic S., Novak N., Jakasa I. (2014). Skin barrier in atopic dermatitis. *Frontiers in Bioscience*.

[16] Talpur R., Bassett R., Duvic M. (2008). Prevalence and treatment of *Staphylococcus aureus* colonization in patients with mycosis fungoides and Sézary syndrome. *British Journal of Dermatology*.

[17] Guenova E., Hoetzenecker W., Rozati S., Levesque M. P., Dummer R., Cozzio A. (2014). Novel therapies for cutaneous T-cell lymphoma: what does the future hold?. *Expert Opinion on Investigational Drugs*.

[18] Miyagaki T., Sugaya M. (2011). Erythrodermic cutaneous T-cell lymphoma: how to differentiate this rare disease from atopic dermatitis. *Journal of Dermatological Science*.

[19] Cather J. C., Vance E. A., Menter M. A. (2002). Diverse cutaneous manifestations associated with a single disease. *Proceedings (Baylor University. Medical Center)*.

[20] Parmentier L., Dürr C., Vassella E., Beltraminelli H., Borradori L., Haneke E. (2010). Specific nail alterations in cutaneous T-cell lymphoma: successful treatment with topical mechlorethamine. *Archives of Dermatology*.

[21] Bi M. Y., Curry J. L., Christiano A. M. (2011). The spectrum of hair loss in patients with mycosis fungoides and Sézary syndrome. *Journal of the American Academy of Dermatology*.

[22] Cook B. E. Jr., Bartley G. B., Pittelkow M. R., Kennedy R. H. (1998). Ophthalmic abnormalities in patients with cutaneous T-cell lymphoma. *Transactions of the American Ophthalmological Society*.

[23] Mehrany K., El-Azhary R. A., Bouwhuis S. A., Pittelkow M. R. (2003). Cutaneous T-cell lymphoma and atopy: is there an association?. *British Journal of Dermatology*.

[24] Rajka G., Winkelmann R. K. (1984). Atopic dermatitis and Sezary syndrome. *Archives of Dermatology*.

[25] Van Haselen C. W., Toonstra J., Preesman A. H., Van Der Putte S. C. J., Bruijnzeel-Koomen C. A. F. M., Van Vloten W. A. (1999). Sezary syndrome in a young man with severe atopic dermatitis. *British Journal of Dermatology*.

[26] Abel E. A., Nickoloff B. J., Shelby D. M., Watson W., Wood G. S. (1986). Tumor stage mycosis fungoides in a patient treated with long-term corticosteroids for asthma and atopic-like dermatitis. *Journal of Dermatologic Surgery and Oncology*.

[27] Vonderheid E. C. (2006). On the diagnosis of erythrodermic cutaneous T-cell lymphoma. *Journal of Cutaneous Pathology*.

[28] Russell-Jones R. (2005). Diagnosing erythrodermic cutaneous T-cell lymphoma. *British Journal of Dermatology*.

[29] LeBoit P. E. (2013). Simulators of cutaneous lymphoma where should our efforts go?. *American Journal of Clinical Pathology*.

[30] Bieber T. (2008). Atopic dermatitis. *New England Journal of Medicine*.

[31] Katsarou A., Armenaka M. C. (2011). Atopic dermatitis in older patients: particular points. *Journal of the European Academy of Dermatology and Venereology*.

[32] Ozkaya E. (2005). Adult-onset atopic dermatitis. *Journal of the American Academy of Dermatology*.

[33] Guenova E., Watanabe R., Teague J. E. (2013). TH2 cytokines from malignant cells suppress TH1 responses and enforce a global TH2 bias in leukemic cutaneous T-cell lymphoma. *Clinical Cancer Research*.

[34] Guenova E., Volz T., Sauer K. (2008). IL-4-mediated fine tuning of IL-12p70 production by human DC. *European Journal of Immunology*.

[35] Vermeer M. H., van Doorn R., Dukers D., Bekkenk M. W., Meijer C. J. L. M., Willemze R. (2001). CD8^+^ T cells in cutaneous T-cell lymphoma: expression of cytotoxic proteins, Fas ligand, and killing inhibitory receptors and their relationship with clinical behavior. *Journal of Clinical Oncology*.

[36] Goteri G., Filosa A., Mannello B. (2003). Density of neoplastic lymphoid infiltrate, CD8+ T cells, and CD1a+ dendritic cells in mycosis fungoides. *Journal of Clinical Pathology*.

[37] Jawed S. I., Myskowski P. L., Horwitz S., Moskowitz A., Querfeld C. (2014). Primary cutaneous T-cell lymphoma (mycosis fungoides and Sézary syndrome): part I. Diagnosis: clinical and histopathologic features and new molecular and biologic markers. *Journal of the American Academy of Dermatology*.

[38] Gjerdrum L. M., Woetmann A., Odum N. (2007). FOXP3+ regulatory T cells in cutaneous T-cell lymphomas: association with disease stage and survival. *Leukemia*.

[39] Krejsgaard T., Odum N., Geisler C., Wasik M. A., Woetmann A. (2012). Regulatory T cells and immunodeficiency in mycosis fungoides and Sézary syndrome. *Leukemia*.

[40] Ni X., Hazarika P., Zhang C., Talpur R., Duvic M. (2001). Fas ligand expression by neoplastic T lymphocytes mediates elimination of CD8^+^ cytotoxic T lymphocytes in mycosis fungoides: a potential mechanism of tumor immune escape?. *Clinical Cancer Research*.

[41] Berger C. L., Tigelaar R., Cohen J. (2005). Cutaneous T-cell lymphoma: malignant proliferation of T-regulatory cells. *Blood*.

[42] Dummer R., Heald P. W., Nestle F. O. (1996). Sézary syndrome T-cell clones display T-helper 2 cytokines and express the accessory factor-1 (interferon-gamma receptor beta-chain). *Blood*.

[43] Krejsgaard T., Ralfkiaer U., Clasen-Linde E. (2011). Malignant cutaneous T-cell lymphoma cells express IL-17 utilizing the Jak3/stat3 signaling pathway. *Journal of Investigative Dermatology*.

[44] Papadavid E., Economidou J., Psarra A. (2003). The relevance of peripheral blood T-helper 1 and 2 cytokine pattern in the evaluation of patients with mycosis fungoides and Sézary syndrome. *British Journal of Dermatology*.

[45] Vowels B. R., Cassin M., Vonderheid E. C., Rook A. H. (1992). Aberrant cytokine production by sezary syndrome patients: cytokine secretion pattern resembles murine Th2 cells. *Journal of Investigative Dermatology*.

[46] Kari L., Loboda A., Nebozhyn M. (2003). Classification and prediction of survival in patients with the leukemic phase of cutaneous T cell lymphoma. *Journal of Experimental Medicine*.

[47] Vowels B. R., Lessin S. R., Cassin M. (1994). Th2 cytokine mRNA expression in skin in cutaneous T-cell lymphoma. *Journal of Investigative Dermatology*.

[48] Rübben A., Kempf W., Kadin M. E., Zimmermann D. R., Burg G. (2004). Multilineage progression of genetically unstable tumor subclones in cutaneous T-cell lymphoma. *Experimental Dermatology*.

[49] Yawalkar N., Ferenczi K., Jones D. A. (2003). Profound loss of T-cell receptor repertoire complexity in cutaneous T-cell lymphoma. *Blood*.

[50] Leung D. Y., Bieber T. (2003). Atopic dermatitis. *The Lancet*.

[51] Gittler J. K., Shemer A., Suarez-Farinas M. (2012). Progressive activation of T(H)2/T(H)22 cytokines and selective epidermal proteins characterizes acute and chronic atopic dermatitis. *Journal of Allergy and Clinical Immunology*.

[52] Biedermann T., Röcken M., Carballido J. M. (2004). TH1 and TH2 lymphocyte development and regulation of TH cell-mediated immune responses of the skin. *Journal of Investigative Dermatology Symposium Proceedings*.

[53] Volz T., Skabytska Y., Guenova E. (2014). Nonpathogenic bacteria alleviating atopic dermatitis inflammation induce IL-10-producing dendritic cells and regulatory Tr1 cells. *Journal of Investigative Dermatology*.

[54] Skabytska Y., Wölbing F., Günther C. (2014). Cutaneous innate immune sensing of toll-like receptor 2-6 ligands suppresses T cell immunity by inducing myeloid-derived suppressor cells. *Immunity*.

[55] Soumelis V., Reche P. A., Kanzler H. (2002). Human epithelial cells trigger dendritic cell−mediated allergic inflammation by producing TSLP. *Nature Immunology*.

[56] Matsumoto M., Ra C., Kawamoto K. (1999). IgE hyperproduction through enhanced tyrosine phosphorylation of janus kinase 3 in NC/Nga mice, a model for human atopic dermatitis. *Journal of Immunology*.

[57] Kataoka Y. (2014). Thymus and activation-regulated chemokine as a clinical biomarker in atopic dermatitis. *Journal of Dermatology*.

[58] Thijs J., Krastev T., Weidinger S. (2015). Biomarkers for atopic dermatitis: a systematic review and meta-analysis. *Current Opinion in Allergy and Clinical Immunology*.

[59] Kim E. J., Hess S., Richardson S. K. (2005). Immunopathogenesis and therapy of cutaneous T cell lymphoma. *The Journal of Clinical Investigation*.

[60] Lu D., Duvic M., Medeiros L. J., Luthra R., Dorfman D. M., Jones D. (2001). The T-cell chemokine receptor CXCR3 is expressed highly in low-grade mycosis fungoides. *American Journal of Clinical Pathology*.

[61] Grewe M., Walther S., Gyufko K., Czech W., Schopf E., Krutmann J. (1995). Analysis of the cytokine pattern expressed in situ in inhalant allergen patch test reactions of atopic dermatitis patients. *Journal of Investigative Dermatology*.

[62] Taha R. A., Leung D. Y. M., Ghaffar O., Boguniewicz M., Hamid Q. (1998). In vivo expression of cytokine receptor mRNA in atopic dermatitis. *Journal of Allergy and Clinical Immunology*.

[63] Kaesler S., Volz T., Skabytska Y. (2014). Toll-like receptor 2 ligands promote chronic atopic dermatitis through IL-4-mediated suppression of IL-10. *Journal of Allergy and Clinical Immunology*.

[64] Kasprzycka M., Zhang Q., Witkiewicz A. (2008). *γ*c-signaling cytokines induce a regulatory T cell phenotype in malignant CD4^+^ T lymphocytes. *Journal of Immunology*.

[65] Krejsgaard T., Gjerdrum L. M., Ralfkiaer E. (2008). Malignant Tregs express low molecular splice forms of FOXP3 in Sézary syndrome. *Leukemia*.

[66] Bagot M., Nikolova M., Schirm-Chabanette F., Wechsler J., Boumsell L., Bensussan A. (2001). Crosstalk between tumor T lymphocytes and reactive T lymphocytes in cutaneous T cell lymphomas. *Annals of the New York Academy of Sciences*.

[67] Jin H. T., Ahmed R., Okazaki T. (2011). Role of PD-1 in regulating T-cell immunity. *Negative Co-Receptors and Ligands*.

[68] Çetinözman F., Jansen P. M., Vermeer M. H., Willemze R. (2012). Differential expression of programmed death-1 (PD-1) in Sézary syndrome and mycosis fungoides. *Archives of Dermatology*.

[69] Samimi S., Benoit B., Evans K. (2010). Increased programmed death-1 expression on CD4+ T cells in cutaneous T-cell lymphoma: implications for immune suppression. *Archives of Dermatology*.

[70] Contassot E., Kerl K., Roques S. (2008). Resistance to FasL and tumor necrosis factor-related apoptosis-inducing ligand-mediated apoptosis in Sézary syndrome T-cells associated with impaired death receptor and FLICE-inhibitory protein expression. *Blood*.

[71] Dereure O., Levi E., Vonderheid E. C., Kadin M. E. (2002). Infrequent Fas mutations but no Bax or p53 mutations in early mycosis fungoides: a possible mechanism for the accumulation of malignant T lymphocytes in the skin. *Journal of Investigative Dermatology*.

[72] Dereure O., Portales P., Clot J., Guilhou J.-J. (2000). Decreased expression of Fas (APO-1/CD95) on peripheral blood CD4^+^ T lymphocytes in cutaneous T-cell lymphomas. *British Journal of Dermatology*.

[73] Wu J., Nihal M., Siddiqui J., Vonderheid E. C., Wood G. S. (2009). Low FAS/CD95 expression by CTCL correlates with reduced sensitivity to apoptosis that can be restored by FAS upregulation. *Journal of Investigative Dermatology*.

[74] Jones C. L., Wain E. M., Chu C.-C. (2010). Downregulation of fas gene expression in sézary syndrome is associated with promoter hypermethylation. *Journal of Investigative Dermatology*.

[75] van Doorn R., Dijkman R., Vermeer M. H., Starink T. M., Willemze R., Tensen C. P. (2002). A novel splice variant of the Fas gene in patients with cutaneous T-cell lymphoma. *Cancer Research*.

[76] Reiss Y., Proudfoot A. E., Power C. A., Campbell J. J., Butcher E. C. (2001). CC chemokine receptor (CCR)4 and the CCR10 ligand cutaneous T cell-attracting chemokine (CTACK) in lymphocyte trafficking to inflamed skin. *Journal of Experimental Medicine*.

[77] Ferenczi K., Fuhlbrigge R. C., Pinkus J. L., Pinkus G. S., Kupper T. S. (2002). Increased CCR4 expression in cutaneous T cell lymphoma. *Journal of Investigative Dermatology*.

[78] Kakinuma T., Sugaya M., Nakamura K. (2003). Thymus and activation-regulated chemokine (TARC/CCL17) in mycosis fungoides: serum TARC levels reflect the disease activity of mycosis fungoides. *Journal of the American Academy of Dermatology*.

[79] Robert C., Kupper T. S. (1999). Inflammatory skin diseases, T cells, and immune surveillance. *New England Journal of Medicine*.

[80] Yagi H., Seo N., Ohshima A. (2006). Chemokine receptor expression in cutaneous T cell and NK/T-cell lymphomas: Immunohistochemical staining and in vitro chemotactic assay. *American Journal of Surgical Pathology*.

[81] Yamaguchi T., Ohshima K., Tsuchiya T. (2003). The comparison of expression of cutaneous lymphocyte-associated antigen (CLA), and Th1- and Th2-associated antigens in mycosis fungoides and cutaneous lesions of adult T-cell leukemia/lymphoma. *European Journal of Dermatology*.

[82] Sokolowska-Wojdylo M., Wenzel J., Gaffal E. (2005). Circulating clonal CLA+ and CD4+ T cells in Sézary syndrome express the skin-homing chemokine receptors CCR4 and CCR10 as well as the lymph node-homing chemokine receptor CCR7. *British Journal of Dermatology*.

[83] Kallinich T., Muche J. M., Qin S., Sterry W., Audring H., Kroczek R. A. (2003). Chemokine receptor expression on neoplastic and reactive T cells in the skin at different stages of mycosis fungoides. *Journal of Investigative Dermatology*.

[84] Narducci M. G., Scala E., Bresin A. (2006). Skin homing of Sézary cells involves SDF-1-CXCR4 signaling and down-regulation of CD26/dipeptidylpeptidase IV. *Blood*.

[85] Capriotti E., Vonderheid E. C., Thoburn C. J., Bright E. C., Hess A. D. (2007). Chemokine receptor expression by leukemic T cells of cutaneous T-cell lymphoma: clinical and histopathological correlations. *Journal of Investigative Dermatology*.

[86] Tendler C. L., Burton J. D., Jaffe J. (1994). Abnormal cytokine expression in Sézary and adult T-cell leukemia cells correlates with the functional diversity between these T-cell malignancies. *Cancer Research*.

[87] Sugaya M. (2010). Chemokines and cutaneous lymphoma. *Journal of Dermatological Science*.

[88] Forssmann U., Uguccioni M., Loetscher P. (1997). Eotaxin-2, a novel CC chemokine that is selective for the chemokine receptor CCR3, and acts like eotaxin on human eosinophil and basophil leukocytes. *Journal of Experimental Medicine*.

[89] Sallusto F., Mackay C. R., Lanzavecchia A. (1997). Selective expression of the eotaxin receptor CCR3 by human T helper 2 cells. *Science*.

[90] Miyagaki T., Sugaya M., Fujita H. (2010). Eotaxins and CCR3 interaction regulates the Th2 environment of cutaneous T-Cell lymphoma. *Journal of Investigative Dermatology*.

[91] Berkman N., Ohnona S., Chung F. K., Breuer R. (2001). Eotaxin-3 but not eotaxin gene expression is upregulated in asthmatics 24 hours after allergen challenge. *American Journal of Respiratory Cell and Molecular Biology*.

[92] Kagami S., Kakinuma T., Saeki H. (2003). Significant elevation of serum levels of eotaxin-3/CCL26, but not of eotaxin-2/CCL24, in patients with atopic dermatitis: serum eotaxin-3/CCL26 levels reflect the disease activity of atopic dermatitis. *Clinical and Experimental Immunology*.

[93] Ebner S., Nguyen V. A., Forstner M. (2007). Thymic stromal lymphopoietin converts human epidermal Langerhans cells into antigen-presenting cells that induce proallergic T cells. *Journal of Allergy and Clinical Immunology*.

[94] Yoo J., Omori M., Gyarmati D. (2005). Spontaneous atopic dermatitis in mice expressing an inducible thymic stromal lymphopoietin transgene specifically in the skin. *Journal of Experimental Medicine*.

[95] Miyagaki T., Sugaya M. (2014). Immunological milieu in mycosis fungoides and Sézary syndrome. *Journal of Dermatology*.

[96] Mollanazar N. K., Smith P. K., Yosipovitch G. (2015). Mediators of chronic pruritus in atopic dermatitis: getting the itch out?. *Clinical Reviews in Allergy and Immunology*.

[97] Sokołowska-Wojdyło M., Gleń J., Zabłotna M. (2013). Association of distinct IL-31 polymorphisms with pruritus and severity of atopic dermatitis. *Journal of the European Academy of Dermatology and Venereology*.

[98] Hartmann K., Wagner N., Rabenhorst A. (2013). Serum IL-31 levels are increased in a subset of patients with mastocytosis and correlate with disease severity in adult patients. *Journal of Allergy and Clinical Immunology*.

[99] Möbs M., Gryzik S., Haidar A., Humme D., Beyer M., Vandersee S. (2014). Analysis of the IL-31 pathway in mycosis fungoides and sézary syndrome. *Archives of Dermatological Research*.

[100] Ong P. Y., Ohtake T., Brandt C. (2002). Endogenous antimicrobial peptides and skin infections in atopic dermatitis. *The New England Journal of Medicine*.

[101] Nomura I., Goleva E., Howell M. D. (2003). Cytokine milieu of atopic dermatitis, as compared to psoriasis, skin prevents induction of innate immune response genes. *The Journal of Immunology*.

[102] Howell M. D., Wollenberg A., Gallo R. L. (2006). Cathelicidin deficiency predisposes to eczema herpeticum. *Journal of Allergy and Clinical Immunology*.

[103] Axelrod P. I., Lorber B., Vonderheid E. C. (1992). Infections complicating mycosis fungoides and sézary syndrome. *The Journal of the American Medical Association*.

[104] Jackow C. M., Cather J. C., Hearne V., Asano A. T., Musser J. M., Duvic M. (1997). Association of erythrodermic cutaneous T-cell lymphoma, superantigen- positive *Staphylococcus aureus*, and oligoclonal T-cell receptor V*β* gene expansion. *Blood*.

[105] Nguyen V., Huggins R. H., Lertsburapa T. (2008). Cutaneous T-cell lymphoma and *Staphylococcus aureus* colonization. *Journal of the American Academy of Dermatology*.

[106] Gilani S. J. K., Gonzalez M., Hussain I., Finlay A. Y., Patel G. K. (2005). *Staphylococcus aureus* re-colonization in atopic dermatitis: beyond the skin. *Clinical and Experimental Dermatology*.

[107] Roll A., Cozzio A., Fischer B., Schmid-Grendelmeier P. (2004). Microbial colonization and atopic dermatitis. *Current Opinion in Allergy and Clinical Immunology*.

[108] Nobbe S., Dziunycz P., Mühleisen B. (2012). IL-31 expression by inflammatory cells is preferentially elevated in atopic dermatitis. *Acta Dermato-Venereologica*.

[109] Sonkoly E., Muller A., Lauerma A. I. (2006). IL-31: a new link between T cells and pruritus in atopic skin inflammation. *Journal of Allergy and Clinical Immunology*.

[110] Sommer V. H., Clemmensen O. J., Nielsen O. (2004). In vivo activation of STAT3 in cutaneous T-cell lymphoma. Evidence for an antiapoptotic function of STAT3. *Leukemia*.

[111] Asadullah K., Döcke W.-D., Haeuler A., Sterry W., Volk H.-D. (1996). Progression of mycosis fungoides is associated with increasing cutaneous expression of interleukin-10 mRNA. *Journal of Investigative Dermatology*.

[112] Krejsgaard T., Willerslev-Olsen A., Lindahl L. M. (2014). Staphylococcal enterotoxins stimulate lymphoma-associated immune dysregulation. *Blood*.

[113] Tokura Y., Yagi H., Ohshima A. (1995). Cutaneous colonization with staphylococci influences the disease activity of Sézary syndrome: a potential role for bacterial superantigens. *British Journal of Dermatology*.

[114] Wong H. K., Mishra A., Hake T., Porcu P. (2011). Evolving insights in the pathogenesis and therapy of cutaneous T-cell lymphoma (mycosis fungoides and sezary syndrome). *British Journal of Haematology*.

[115] Guenova E., Skabytska Y., Hoetzenecker W. (2015). IL-4 abrogates T_H_17 cell-mediated inflammation by selective silencing of IL-23 in antigen-presenting cells. *Proceedings of the National Academy of Sciences of the United States of America*.

[116] Dummer R., Rozati S., Guenova E., Cozzio A. (2013). Less can be more: the impact of chemotherapy on cutaneous T-cell lymphomas. *Future Oncology*.

[117] Trautinger F., Knobler R., Willemze R. (2006). EORTC consensus recommendations for the treatment of mycosis fungoides/Sézary syndrome. *European Journal of Cancer*.

[118] Sidbury R., Davis D. M., Cohen D. E. (2014). Guidelines of care for the management of atopic dermatitis: Section 3. Management and treatment with phototherapy and systemic agents. *Journal of the American Academy of Dermatology*.

[119] Olsen E. A., Rook A. H., Zic J. (2011). Sézary syndrome: immunopathogenesis, literature review of therapeutic options, and recommendations for therapy by the United States Cutaneous Lymphoma Consortium (USCLC). *Journal of the American Academy of Dermatology*.

[120] Guitart J., Wickless S. C., Oyama Y. (2002). Long-term remission after allogeneic hematopoietic stem cell transplantation for refractory cutaneous T-cell lymphoma. *Archives of Dermatology*.

[121] Querfeld C., Rosen S. T., Kuzel T. M. (2005). Long-term follow-up of patients with early-stage cutaneous T-cell lymphoma who achieved complete remission with psoralen plus UV-A monotherapy. *Archives of Dermatology*.

[122] Mansouri Y., Guttman-Yassky E. (2015). Immune pathways in atopic dermatitis, and definition of biomarkers through broad and targeted therapeutics. *Journal of Clinical Medicine*.

[123] Lundin J., Hagberg H., Repp R. (2003). Phase 2 study of alemtuzumab (anti-CD52 monoclonal antibody) in patients with advanced mycosis fungoides/Sézary syndrome. *Blood*.

[124] Clark R. A., Watanabe R., Teague J. E. (2012). Skin effector memory T cells do not recirculate and provide immune protection in alemtuzumab-treated CTCL patients. *Science Translational Medicine*.

[125] Thursky K. A., Worth L. J., Seymour J. F., Miles Prince H., Slavin M. A. (2006). Spectrum of infection, risk and recommendations for prophylaxis and screening among patients with lymphoproliferative disorders treated with alemtuzumab. *British Journal of Haematology*.

[126] Gibson H. M., Mishra A., Chan D. V., Hake T. S., Porcu P., Wong H. K. (2013). Impaired proteasome function activates GATA3 in T cells and upregulates CTLA-4: relevance for Sézary syndrome. *The Journal of Investigative Dermatology*.

[127] Wong H. K., Wilson A. J., Gibson H. M. (2006). Increased expression of CTLA-4 in malignant T cells from patients with mycosis fungoides - Cutaneous T-cell lymphoma. *Journal of Investigative Dermatology*.

[128] Patnaik A., Kang S. P., Rasco D. (2015). Phase i study of pembrolizumab (MK-3475; Anti-PD-1 monoclonal antibody) in patients with advanced solid tumors. *Clinical Cancer Research*.

[129] Chvatchko Y., Hoogewerf A. J., Meyer A. (2000). A key role for CC chemokine receptor 4 in lipopolysaccharide-induced endotoxic shock. *The Journal of Experimental Medicine*.

[130] Duvic M., Pinter-Brown L. C., Foss F. M. (2015). Phase 1/2 study of mogamulizumab, a defucosylated anti-CCR4 antibody, in previously treated patients with cutaneous T-cell lymphoma. *Blood*.

[131] Ni X., Langridge T., Duvic M. (2015). Depletion of regulatory T cells by targeting CC chemokine receptor type 4 with mogamulizumab. *OncoImmunology*.

[132] Sugaya M., Morimura S., Suga H. (2015). CCR4 is expressed on infiltrating cells in lesional skin of early mycosis fungoides and atopic dermatitis. *The Journal of Dermatology*.

[133] Piancatelli D., Bellotta L., Del Beato T., Duse M., Della Penna M. R. (2008). Total il-12 levels are increased in paediatric atopic dermatitis: correlations with age and disease severity. *International Journal of Immunopathology and Pharmacology*.

[134] Kondo N., Matsui E., Kaneko H. (2001). Reduced interferon-*γ* production and mutations of the interleukin-12 receptor *β*
_2_ chain gene in atopic subjects. *International Archives of Allergy and Immunology*.

[135] Eun J. K., Won M. L., Jung S. H., Nam H. R., Dong S. J., Jae R. K. (2006). mRNA expression and RNA editing (2451 C-to-U) of IL-12 receptor *β*2 in adult atopic patients. *Journal of Korean Medical Science*.

[136] Rook A. H., Wood G. S., Yoo E. K. (1999). Interleukin-12 therapy of cutaneous T-cell lymphoma induces lesion regression and cytotoxic T-cell responses. *Blood*.

[137] Wasik M. A. (2015). IL-13 as a novel growth factor in CTCL. *Blood*.

[138] Geskin L. J., Viragova S., Stolz D. B., Fuschiotti P. (2015). Interleukin-13 is overexpressed in cutaneous T-cell lymphoma cells and regulates their proliferation. *Blood*.

[139] Hanania N. A., Noonan M., Corren J. (2015). Lebrikizumab in moderate-to-severe asthma: pooled data from two randomised placebo-controlled studies. *Thorax*.

[140] Beck L. A., Thaçi D., Hamilton J. D. (2014). Dupilumab treatment in adults with moderate-to-severe atopic dermatitis. *New England Journal of Medicine*.

[141] Hamilton J. D., Suárez-Fariñas M., Dhingra N. (2014). Dupilumab improves the molecular signature in skin of patients with moderate-to-severe atopic dermatitis. *Journal of Allergy and Clinical Immunology*.

[142] Tsianakas A., Luger T. A. (2015). The anti-IL-4 receptor alpha antibody dupilumab: facing a new era in treating atopic dermatitis. *Expert Opinion on Biological Therapy*.

[143] Sigurdsson V., Toonstra J., Bihari I. C., Bruijnzeel-Koomen C. A. F. M., van Vloten W. A., Thepen T. (2000). Interleukin 4 and interferon-*γ* expression of the dermal infiltrate in patients with erythroderma and mycosis fungoides. An immuno-histochemical study. *Journal of Cutaneous Pathology*.

[144] Woo J. H., Lee Y.-J., Neville D. M., Frankel A. E. (2010). Pharmacology of anti-CD3 diphtheria immunotoxin in CD3 positive T-cell lymphoma trials. *Methods in Molecular Biology*.

[145] Rook A. H. (2012). The beauty of TLR agonists for CTCL. *Blood*.

[146] Huen A. O., Rook A. H. (2014). Toll receptor agonist therapy of skin cancer and cutaneous T-cell lymphoma. *Current Opinion in Oncology*.

[147] Martínez-González M. C., Verea-Hernando M. M., Yebra-Pimentel M. T., Del Pozo J., Mazaira M., Fonseca E. (2008). Imiquimod in mycosis fungoides. *European Journal of Dermatology*.

[148] Onsun N., Ufacik H., Kural Y., Topçu E., Somay A. (2005). Efficacy of imiquimod in solitary plaques of mycosis fungoides. *International Journal of Tissue Reactions*.

[149] Deeths M. J., Chapman J. T., Dellavalle R. P., Zeng C., Aeling J. L. (2005). Treatment of patch and plaque stage mycosis fungoides with imiquimod 5% cream. *Journal of the American Academy of Dermatology*.

[150] Kim Y. H., Girardi M., Duvic M. (2010). Phase i trial of a Toll-like receptor 9 agonist, PF-3512676 (CPG 7909), in patients with treatment-refractory, cutaneous T-cell lymphoma. *Journal of the American Academy of Dermatology*.

[151] Kim Y. H., Gratzinger D., Harrison C. (2012). In situ vaccination against mycosis fungoides by intratumoral injection of a TLR9 agonist combined with radiation: a phase 1/2 study. *Blood*.

[152] Sagiv-Barfi I., Kohrt H. E., Burckhardt L., Czerwinski D. K., Levy R. (2015). Ibrutinib enhances the antitumor immune response induced by intratumoral injection of a TLR9 ligand in mouse lymphoma. *Blood*.

[153] Fujita H. (2013). The role of IL-22 and Th22 cells in human skin diseases. *Journal of Dermatological Science*.

[154] Miyagaki T., Sugaya M., Suga H. (2011). IL-22, but not IL-17, Dominant environment in cutaneous T-cell lymphoma. *Clinical Cancer Research*.

[155] Kuppala M. B., Syed S. B., Bandaru S., Varre S., Akka J., Mundulru H. P. (2012). Immunotherapeutic approach for better management of cancer—role of IL-18. *Asian Pacific Journal of Cancer Prevention*.

[156] Amo Y., Ohta Y., Hamada Y., Katsuoka K. (2001). Serum levels of interleukin-18 are increased in patients with cutaneous T-cell lymphoma and cutaneous natural killer-cell lymphoma. *The British Journal of Dermatology*.

[157] Yamanaka K.-I., Clark R., Dowgiert R. (2006). Expression of interleukin-18 and caspase-1 in cutaneous T-cell lymphoma. *Clinical Cancer Research*.

[158] Park H., Byun D., Kim T. S. (2001). Enhanced IL-18 expression in common skin tumors. *Immunology Letters*.

[159] Robertson M. J., Kline J., Struemper H. (2013). A dose-escalation study of recombinant human interleukin-18 in combination with rituximab in patients with non-hodgkin Lymphoma. *Journal of Immunotherapy*.

[160] Aral M., Arican O., Gul M. (2006). The relationship between serum levels of total IgE, IL-18, IL-12, IFN-*γ* and disease severity in children with atopic dermatitis. *Mediators of Inflammation*.

[161] Zedan K., Rasheed Z., Farouk Y. (2015). Immunoglobulin E, interleukin-18 and interleukin-12 in patients with atopic dermatitis: correlation with disease activity. *Journal of Clinical and Diagnostic Research*.

[162] Konishi H., Tsutsui H., Murakami T. (2002). IL-18 contributes to the spontaneous development of atopic dermatitis-like inflammatory skin lesion independently of IgE/stat6 under specific pathogen-free conditions. *Proceedings of the National Academy of Sciences of the United States of America*.

[163] Mehra T., Ikenberg K., Moos R. M. (2015). Brentuximab as a treatment for CD30^+^ mycosis fungoides and Sézary syndrome. *JAMA Dermatology*.

[164] Chiarle R., Podda A., Prolla G., Gong J., Thorbecke G. J., Inghirami G. (1999). CD30 in normal and neoplastic cells. *Clinical Immunology*.

[165] Petersen D. L., Krejsgaard T., Berthelsen J. (2014). B-lymphoid tyrosine kinase (Blk) is an oncogene and a potential target for therapy with dasatinib in cutaneous T-cell lymphoma (CTCL). *Leukemia*.

